# A Novel Observational Method for Assessing Acute Responses to Cannabis: Preliminary Validation Using Legal Market Strains

**DOI:** 10.1089/can.2017.0038

**Published:** 2018-03-01

**Authors:** L. Cinnamon Bidwell, Raeghan Mueller, Sophie L. YorkWilliams, Sarah Hagerty, Angela D. Bryan, Kent E. Hutchison

**Affiliations:** ^1^ Institute of Cognitive Science, University of Colorado Boulder, Boulder, Colorado.; ^2^ Department of Psychology and Neuroscience, University of Colorado Boulder, Boulder, Colorado.

**Keywords:** cannabis, CBD, harm reduction, subjective effects, THC

## Abstract

**Background:** The development of novel cannabis research methods that are compatible with current federal regulations is imperative to conduct studies of the effects of legal market cannabis. There is very little research on higher strength, higher Δ9-tetrahydrocannabinol (THC), which has become increasingly available since legalization. Research on strains containing cannabidiol (CBD), a second primary, but nonpsychotomimetic, cannabinoid, is very limited.

**Materials and Methods:** Using a novel observational methodology, regular cannabis users were asked to use one of two legal market cannabis strains that they purchased from a local dispensary (one strain containing 8% THC and 16% CBD (THC+CBD) and one containing a 17% THC concentration, but no CBD (THC). After using their suggested cannabis strain as they typically would for a 3-day period, participants returned to the laboratory immediately after their final use. Measures included a blood draw to measure cannabinoid blood levels and circulating cytokines, self-reported subjective drug effects, and verbal recall memory.

**Results:** Analysis of CBD/THC concentration levels in the blood following the 3-day strain manipulation suggests that all, but one participant (*n*=23/24) followed instructions and used their assigned strain. Individuals in the THC group (*n*=11) smoked no more than their usual amount, and participants who used the THC+CBD (*n*=12) strain smoked more than their reported usual amount, but did not have significantly different THC+metabolite blood levels from the THC group. The THC+CBD strain was also associated with less desire to smoke, lower levels of subjective drug effects, and lower levels of circulating cytokines (TNF-α, IL-6, and IL-1β) immediately after use.

**Conclusions:** Initial results support the feasibility of this novel observational methodology involving brief manipulation of strain use. Preliminary findings indicate that participants may self-titrate cannabis use based on cannabinoid concentration and the THC+CBD strain was associated with lower levels of cannabis craving, subjective intoxication, and circulating cytokines.

## Introduction

Over the last several years, there have been enormous changes concerning the public acceptance of cannabis. Twenty-nine states and the District of Columbia (DC) have legalized medicinal use, while eight states and DC have legalized recreational use. The State of Colorado Marijuana Enforcement Division (MED) reports that more than 825,000 cannabis plants were harvested monthly by the middle of 2016 with ∼300,000 pounds of flower sold in 2016.^1^ (“*Flower”* refers to part of the plant material that is commonly smoked and contains the highest cannabinoid concentration.) Considering how much has already changed, externally valid scientific data that can inform public policy and consumer decisions regarding cannabis are critically needed.

Studies dating back to the 1970s have found that cannabis, a dose-dependent effect of Δ9-tetrahydrocannabinol (THC) in particular, acutely increases subjective drug effects such as “high” and “liking,”^2–6^ even when using a “balanced placebo” design.^7,8^ In addition, cannabis produces acute cognitive impairment, especially relating to memory and attention during intoxication (for review see Refs.^9,10^), although regular users may not show these acute decrements in performance.^9^ Research to date has used low-strength government-grown cannabis (THC ranging from 3% to 6%) that lacks other key cannabinoids (CBD close to 0%) and has been administered in tightly controlled laboratory environments, all of which maximize internal validity, but compromise external validity. Currently, the THC strength of recreational cannabis in Colorado can exceed 25%, and the strength of CBD comes close to 25% in some strains.^11^ However, federal regulations do not permit researchers, even those in states with legal cannabis markets, to study such state-regulated cannabis products in a controlled laboratory environment. Thus, there is currently limited or no data on how the varying strengths of cannabis products, which are readily available across the United States, affect use and intoxication levels. We sought to address this gap in the literature by developing and validating a design that could directly examine the effects of self-administration of cannabis in strengths and concentrations commonly available in states like Colorado.

The other important limitation of previous controlled studies on the effects of acute cannabis use is that they often ignore other cannabinoids such as CBD, which may influence the effects of THC. Some analyses from human-based controlled laboratory studies have suggested that CBD may attenuate the negative effects of THC on intoxication,^12^ reward, and cognition,^13–17^ and may also attenuate the “euphoric” subjective effects of THC.^18^ For example, a human-based controlled laboratory study comparing capsules containing placebo, THC (0.5 mg/kg), CBD (1 mg/kg), or a mixture of CBD (1 mg/kg) and THC (0.5 mg/kg) showed that CBD attenuated anxiety symptoms, but did not alter THC's effects on heart rate.^13^ Other studies have examined the effect of pretreatment with oral CBD before THC administration. In one study, subjects were given an oral dose of CBD (600 mg) or placebo 210 min ahead of an intravenous injection of THC (1.5 mg). Those in the CBD group reported less positive psychotic symptoms and greater episodic memory compared to the placebo group.^14^ These studies indicate that cannabinoids such as CBD may interact with the effects of THC.

Consistent with the above, most of the work in the United States on CBD has involved the oral administration of CBD (and often THC) in synthetic form. Studies that focus on the effects of coadministration of THC and CBD on subjective drug reward effects have largely concluded that even high doses of oral synthetic CBD (0, 200, 400, and 800 mg) do not impact the subjective effects of feeling “high” from smoked THC.^19^ However, one study employing a naturalistic design to observe the subjective effects of subjects' chosen cannabis strain, suggested differently. In this study, subjects were asked to smoke their desired strain as they normally would on a typical day.^18^ Those subjects who used a higher CBD:THC strain self-reported lower rewarding effects compared to those using a lower CBD:THC strain. Interestingly, even though there was variation in THC concentrations across the strains, the THC levels detected in saliva samples were similar across the strain groups, suggesting that, when allowed to self-administer naturalistically, individuals titrate up to similar THC saliva levels regardless of THC strength.

Furthermore, pharmacokinetic studies suggest differing dose–response curves when CBD is administered in isolation versus through a CBD-based plant-derived extract that also contains low levels of other phytocannabinoids.^20^ Thus, there may be important differences when assessing the impact of coadministration of these cannabinoids on subjective drug effects when they are used naturalistically, for example, self-administered and smoked in their plant form. In the context of validating a design that can assess the effects of legal market cannabis, we also sought to conduct a preliminary test of the combined effects of THC and CBD when self-administered in plant (i.e., flower) form, the most common route of administration, and when randomly assigning individuals to strains that contain cannabinoid strengths that are consistent with those available on the legal market.

Finally, THC and CBD also seem to have important, but differential impacts on immune system function and inflammation, both peripherally and centrally, which is highly relevant to stress response and the pathophysiology of a number of medical and mental health disorders that overlap with cannabis use; for a review, see Ref. 21. A number of studies have suggested that both THC and CBD modulate proinflammatory cytokines, including TNF-α, IL-6, and IL-1β.^21^ Although both THC and CBD have anti-inflammatory effects, rodent models suggest that this may be particularly true for CBD,^22,23^ where treatment with CBD modulates cytokine immune responses across a number of animal models of human disease states.^24,25^ However, human data are sorely lacking regarding the acute impact of CBD-containing strains on immune responses, which we address as an exploratory aim in this study.

In summary, there is a need for novel designs that allow the study of the effects of legal market cannabis products. However, given the federal legal status of cannabis, this poses a critical research challenge. The primary aim of this pilot study was to validate a novel method that would allow examination of the acute effects of legal market cannabis. The secondary aim was to examine the impact of THC at commonly available strengths with and without the presence of CBD. We hypothesized that the use of a CBD-containing strain might mitigate the intoxicating effects associated with THC. Our alternative hypothesis was that individuals may simply use more of the THC+CBD strain as a means of titrating to their desired level of THC consumption, and therefore achieve similar levels of intoxication and impairment.

To this end, we examined the effects two common strains: one with the average strength of THC in Colorado (Blueberry Diesel, 17% THC, 0% CBD; THC) and one strain that had lower THC, but higher CBD (Cannatonic, 8% THC, 16% CBD; THC+CBD), on measures associated with cannabis use and impairment (quantity consumed, subjective craving and intoxication effects, and verbal recall memory function). In addition to assigning participants to a particular strain group, we employed objective measures of drug exposure by quantifying our primary cannabinoids (THC and CBD) and their relevant metabolites in blood to verify adherence to strain and account for individual variation in use and metabolism. In an exploratory aim, we sought to examine the impact of the two strains on inflammatory stress responses through circulating cytokines.

## Materials and Methods

### Participants

The study was approved by the University of Colorado IRB and legal counsel. Written informed consent was obtained from each participant. Participants (24 male cannabis users aged between 21 and 34 years) were recruited from advertisements posted in local dispensaries and from social media outlets. Participants were screened to exclude daily cigarette smokers, noncannabis drug or alcohol disorder, psychotic symptoms or bipolar disorder, those with neurological or cardiovascular illnesses, and those who had had a serious medical illness within the past 6 months. Participants had to live within 10–15 min drive from the laboratory. In addition, they were required to test negative on toxicology screening (conducted at baseline) for illicit substances (opiates, benzodiazepines, cocaine, and amphetamines) and have a blood alcohol level assessed by breathalyzer of 0.00. Inclusionary criterion included current use of cannabis (at least four times per month for the last 6 months).

### Procedure

The study involved two appointments at our research facility: a baseline assessment and an experimental session immediately after subjects had used cannabis. Before their baseline assessment, subjects were asked to abstain from drinking alcohol and using cannabis for 24 h, and using caffeine or cigarettes for 2 h. Blood was drawn by a trained phlebotomist for quantification of cannabinoids and inflammatory markers, and participants completed a series of questionnaires that captured basic demographic information, past and current substance use, cannabis-specific patterns, and behaviors, personality traits, and mood. In addition, verbal recall memory was assessed by the International Shopping List Task (ISLT).^26^

Upon completion of the baseline appointment, participants were randomly assigned to one of two strains and were asked to acquire and use the strain *ad libitum* over the next 3 days. Participants were asked to use either (1) a common strain with high THC content and little-to-no CBD content (THC ∼17%; CBD 0%; the THC strain) or (2) a strain that contains both THC and CBD (THC ∼8%; CBD ∼16%; the THC+CBD strain). The THC strain was selected because it had the average THC level for Colorado dispensary strains^11^ and the THC+CBD strain was selected because it contained the highest CBD level available at that time. Blood levels provided the primary assessment of THC exposure, thereby accounting for differences in THC levels across the strains as well as individual smoking topography and metabolism.

Participants were given instructions to purchase their typical quantity of either strain “1” or strain “2” from a local dispensary that agreed to assist in the study. While participants had access to specific information regarding the cannabinoid content of their assigned strain, the research assistants interacting with participants were blind to the cannabinoid content of the strains. The participants were not given any instructions on how much of the assigned cannabis strain to purchase, the amount they should use, or the method of consumption. Instead, participants were asked to use the assigned cannabis strain as they normally would until their next scheduled visit (the experimental session), which was ∼3 days later. On the third day, the experimental session was held, and participants used the assigned strain one last time, immediately before being picked up by study staff in a university pool vehicle. After arriving at the facility within 15 min postcannabis use, a blood sample was immediately collected to verify that participants used the correct strain and to quantify drug exposure through levels of THC, CBD, and relevant metabolites in the blood. Participants then completed subjective drug effect and craving questionnaires and the ISLT verbal recall memory test.

### Measures

#### Demographic and background information

Demographic information, including age, cannabis and other substance use history, and current and past anxiety and depression symptomatology, was collected at baseline. Information on the demographic, psychiatric comorbidity, and substance use characteristics of participants are reported in Table 1.

**Table 1.** **Baseline Participant Characteristics and Blood Biomarkers by Strain Group**

	THC strain *n*=11		THC+CBD strain *n*=12			
	*M* or *N*	SD or %	*M* or *N*	SD or %	*t* or χ^2^	*p*
Age	25.1	4.20	23.8	4.20	−1.6	0.13
Number of alcohol drinking days^a^	5.82	4.21	9.83	9.85	1.25	0.23
Number of cannabis smoking days^a^	21.63	8.17	24.33	8.33	0.78	0.44
Average daily cannabis use^b^	0.55	0.77	0.79	0.99	0.67	0.51
Verbal recall at baseline^c^	9.36	1.21	10.09	1.44	−1.28	0.22
Inflammatory plasma biomarkers						
Cytokine average (IL-1β, IL-6, and TNF-α) (ng/mL) at baseline	4.97	7.94	2.94	3.95	−0.78	0.44
Cannabinoid plasma biomarkers						
Baseline (ng/mL)						
THC	1.85	2.36	1.92	2.27	0.06	0.30
CBD	0.0	0.0	0.0	0.0	—	—
THC-COOH	19.95	17.89	36.32	46.65	1.13	0.28
THC-OH	0.2	0.66	0	0	−1	0.34
Average THC+metabolites	7.33	6.52	12.74	16.18	1.07	0.30
Experimental session (Time 2) (ng/mL)						
THC	16.35	14.05	11.97	13.45	−0.772	0.45
CBD	0	0	12.5	9.70	4.32	<0.0001
THC-COOH	45.11	37.55	52.92	64.02	0.36	0.72
THC-OH	3.35	3.67	2.58	3.24	−0.53	0.60
Average THC+metabolites	21.6	17.61	22.49	26.49	0.10	0.93
CBD/THC ratio	0	0	1.33	0.41	10.13	<.0001

^a^ In the past 30 days as determined by Timeline Follow-Back Score.

^b^ Grams smoked per day.

^c^ Correct delayed verbal working memory response from the CogState International Shopping List Task.

THC strain condition smoked a high THC cannabis strain; THC+CBD strain condition smoked a strain containing both THC and CBD.

#### Cannabis and other substance use: frequency and quantity

A Timeline Follow-Back (TLFB) was used to assess daily substance use for the 30 days before the baseline session and as an additional method of verifying user status. The TLFB is a calendar-assisted structured interview that provides the subject with temporal cues to increase the accuracy of recall. This interviewer-administered instrument has demonstrated test-retest reliability and validity. The interviewer conducting the TLFB recorded alcohol use, tobacco use, use of illegal drugs, and recreational use of prescription drugs such as Adderall, Ritalin, and Vicodin. We have modified our TLFB procedure to estimate both the frequency of cannabis use as well as amount used per sitting, day, and week using visual stimuli (e.g., actual size pictures of cannabis measured at 0.10, 0.15, and 0.25 g). Participants were also asked about the administration method (e.g., smoked, vaporized, or consumed in edible form). During the experimental session, subjects were asked to complete a shorter, 7-day TLFB. This second TLFB provided a self-reported estimate of amount used on the days immediately before the experimental session.

#### Subjective effects of cannabis

Participants completed a modified Profile of Mood States (POMS)^27,28^ at each session. In addition to the standard POMS items, the following two questions (rated on a scale from 1 to 5 [1=not at all, 5=extremely]) were included as primary measures of the subjective effects of cannabis: “How mentally stoned do you feel right now?” and “How physically stoned do you feel right now?” They also answered the question “How much do you desire marijuana right now?” on a scale from 0 and 100 (0=not at all, 100=the strongest feeling possible).

#### Verbal delayed recall

To compare performance in verbal learning and memory at baseline versus while under the influence of cannabis, the ISLT was administered at both the baseline and experimental session. The ISLT computer software program consists of a 12-item shopping list that was read out loud to the participant by the experimenter. The participant was never shown the words on the computer screen. After all of the words were read to the participant, the participant was then asked to recall as many words as he remembers. The list of words was presented again in the same order two additional times. After 30 min have passed, a delayed recall trial was administered and participants were asked to recall the list one last time. Participants were read a different shopping list at each session.

#### Blood levels of cannabinoids

Venous blood was collected in a 10 mL EDTA blood collection tube and immediately processed. Plasma was separated from erythrocytes by centrifugation at 400 *g* for 15 min, transferred to a fresh microcentrifuge tube, and stored at −80°C. Plasma samples were sent to iC42 Clinical Research and Development (Department of Anesthesiology) on the Anschutz Medical Campus at the University of Colorado Denver. Six cannabinoids were quantified in the blood (THC, THC-COOH, THC-OH, CBD, and CBN) using validated high-performance liquid chromatography/mass spectroscopy (API5500) in MRM mode.^29^

#### Circulating cytokines

The remaining plasma was used to detect circulating TNF-α, IL-6, and IL-1β levels in the blood. These were measured on a multiplex ELISA^30–32^ following the manufacturer's instructions (Aushon Biosystems, Billerica, MA). As expected, TNF-α, IL-6, and IL-1β expression levels were strongly correlated with one another (*r's*=0.90–0.96). Thus, an average of the three cytokine markers was used as the dependent measure of peripheral inflammation.

### Statistical analyses

The strain groups (THC: *n*=11 and THC+CBD: *n*=12) were compared on baseline variables, using Pearson's chi-square tests for discrete variables and *t*-tests for continuous variables. To test our primary aim of assessing the validity and feasibility of our novel methodology, we examined adherence to strain assignment through blood levels of THC, CBD, and primary relevant metabolites in the two strain groups at the start of the experimental session. Given the rapid rate at which THC is metabolized^33,34^ and the length of time it took to transport participants to the laboratory following their use, THC exposure was calculated by averaging blood levels of THC and its primary metabolites, THC-COOH and THC-OH (THC+metabolite level).

Next, we ran multiple regression models to examine whether a CBD-containing strain (THC+CBD) would be associated with lower scores on measures related to cannabis use and intoxication. Specifically, we tested the interaction of strain type (THC vs. THC+CBD) and level of THC+metabolites on (1) craving/desire to smoke more cannabis; (2) subjective intoxication (2a. feeling mentally stoned and 2b. feeling physically stoned), and (3) cognitive impairment, measured by number of errors on the delayed (30 min) verbal recall test. Equations were as below, with mean-centered THC+metabolite exposure levels and contrast-coded strain type:

\begin{align*}Acute \ Effects \ Measur{e_i} = { \beta _0} + { \beta _1}THCleve{l_i} + { \beta _2}Strai{n_i} \\+ { \beta _3}THClevel{s_i}*Strai{n_i} + { \varepsilon _i}\end{align*}

Finally, we tested our exploratory aim related to possible anti-inflammatory effects of THC and CBD. Given that some animal data suggest that CBD may be particularly anti-inflammatory,^24,25^ we examined the impact of strain assignment on circulating cytokines using an ANCOVA, comparing the average of all three cytokines (TNF-α, IL-6, and IL-1β) at the experimental session between the strain groups, while covarying average baseline cytokine levels.

## Results

### Validity of approach

Our primary aim was to assess the validity and feasibility of our novel observational methodology. We first examined whether cannabinoid blood levels on the day of the postcannabis-use experimental session (Time 2) were indicative of each participant adhering to their assigned strain. Toward this end, we examined THC and CBD blood levels and the CBD:THC ratio at Time 2 across individuals (See Table 1 for cannabinoid blood levels across both time points). All but one individual demonstrated a blood:cannabinoid ratio that matched the CBD:THC cannabinoid ratio of their assigned strain. That is, one individual reported using the CBD strain, but did not have detectable CBD in his blood nor did he have the correct ratio of CBD:THC. Thus, our analyses indicated strong strain adherence across our participants. The single subject whose blood results did not match his assigned strain was excluded from the analyses, resulting in a final sample of 23 participants.

### Impact of cannabis strain on cannabis use

With regard to cannabis use, individuals who received the THC strain smoked the same amount on the experimental day, which they normally smoked. However, individuals who received the THC+CBD strain reported an increase from a mean of 0.259–0.380 g (*p*<0.05, *d*=0.7) on the TLFB. Despite differences in amount used, the THC-only group and the THC+CBD group did not have significantly different blood levels across THC, THC-COOH, and THC-OH. (However, an examination of effect sizes suggested that the THC-only strain group may have had higher blood levels of THC, but this was not significant due to low power [*d*=0.32]). Thus, it appeared that, although the participants who received the THC+CBD strain may have been compensating by smoking more, this did not result in significantly higher THC or THC metabolite blood levels experienced by the THC strain group.

### Impact of cannabis strain on intoxication: subjective drug effects and verbal recall memory

**FIG. 1.**
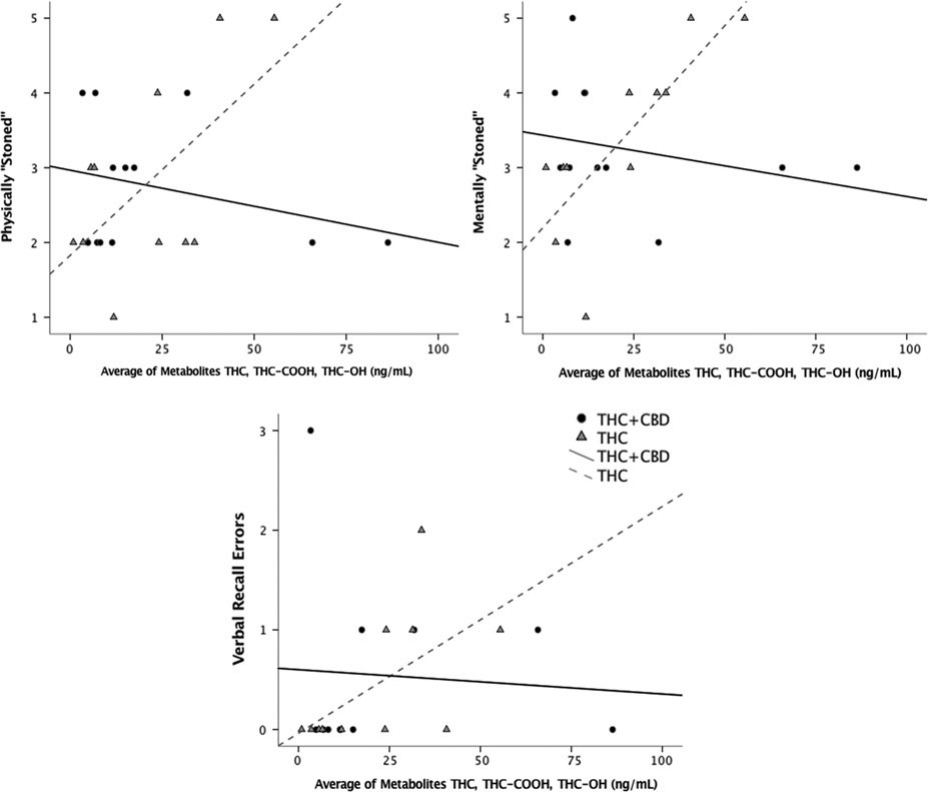
The THC+CBD strain was associated with lower levels of THC-related subjective intoxication and cognitive impairment. Scatter plots show the associations among THC+metabolite blood levels and cannabis intoxication measures at Time 2 (**Panel 1**: feeling mentally stoned, **Panel 2**: physically stoned, and **Panel 3**: verbal recall working memory errors) across the two strain groups (THC; *n*=11 and THC+CBD; *n*=12). Results suggest a positive relationship with THC+metabolite blood levels and intoxication outcomes in the THC-only strain and no significant relationship with THC+metabolite blood levels in the CBD-containing strain (THC+CBD).

Our models revealed higher levels of our three subjective drug effect measures in those assigned to the THC-only strain. At the average THC+metabolite level, use of the THC+CBD strain was associated with a lower desire to smoke more (*p*<0.05; *d*=0.194), and with feeling less intoxicated (less mentally stoned [*p*<0.005; *d*=0.394] and less physically stoned [*p*<0.02; *d*=0.267]) than use of the THC strain (Fig. 1).

*Post hoc* correlations examining simple effects among average THC+metabolite blood levels and subjective effect measures suggested a strong correlation between higher THC in the blood and subjective effects in the THC strain group (desire: *r*=0.60, *p*<0.05; mentally stoned; *r*=0.79, *p*<0.005; and physically stoned: *r*=0.61, *p*<0.05), whereas no strong relationship was detected among average THC+metabolite blood levels and subjective drug effects in the THC+CBD strain group (desire: *r*=−0.30, *p*>0.05; mentally stoned; *r*=−0.25, *p*>0.05; and physically stoned: *r*=−0.29, *p*>0.05). While we did not find a significant interaction between strain type and number of errors on the verbal delay recall test, its effect size (*d*=0.105) suggests that our sample may be underpowered to detect this effect.

Furthermore, *post hoc* correlations among average THC+metabolite blood levels and errors on the verbal delay recall test suggest a correlation between higher THC in the blood and verbal recall errors in the THC strain group (*r*=0.60, *p*<0.05), whereas no strong relationship was detected among THC+metabolite blood levels and verbal recall errors in the THC+CBD strain group (*r*=−0.07, *p*>0.05). Although a larger, fully powered sample is needed to draw strong conclusions about the relationship between strain, THC blood level, subjective effects, and delayed verbal recall, these data provide preliminary evidence that the relationship between THC+metabolite blood levels and increased impairment was reduced in the participants using the CBD-containing strain.

### Validation of cannabinoid blood levels as a predictor of acute effects

To address the potentially confounding effects of cannabis tolerance and cannabis dosage before the experimental session, we ran *post hoc* models of the above regressions, including participants' estimates of amount consumed (1) during a typical usage session (a proxy for tolerance) and (2) during the usage session immediately preceding their appointment (a self-reported measure of cannabis exposure). The interaction results remained significant in these models when either tolerance or self-reported amount used on the session day was included in the model together or separately. Thus, neither regular use patterns nor self-reported amount used before the experimental session were significant predictors of outcomes over and above cannabinoid blood levels. This suggests that THC+metabolite blood levels provide a robust assessment of pharmacological exposure that may be more directly associated with acute responses in regular users then self-reported use patterns.

### Exploratory tests of the impact of cannabinoids on circulating cytokines

**FIG. 2.**
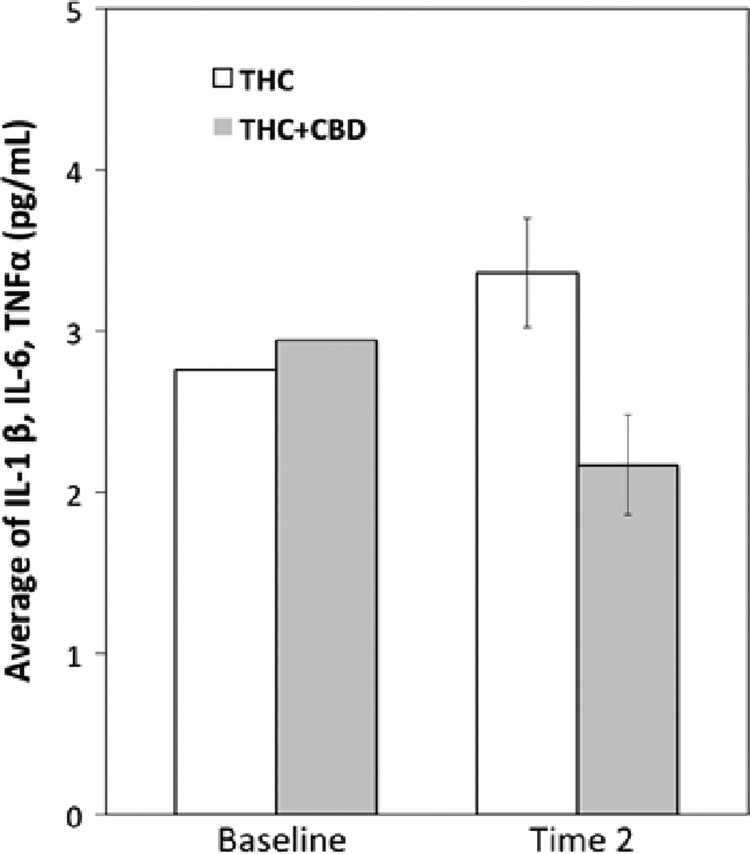
The average plasma concentration of three proinflammatory cytokines (IL-1β, IL-6, and TNF-α) at Baseline and Time 2 for the two strain conditions. THC-only strain group showed a higher average plasma concentration of the three cytokines compared to THC+CBD group. Error bars indicate standard error.

To test the impact of use a CBD-containing strain (THC+CBD) on circulating cytokine levels (average of TNF-α, IL-6, and IL-1β), we ran a one-way ANCOVA comparing the average of all three cytokines (TNF-α, IL-6, and IL-1β) between groups after use of their assigned strain, covarying the average baseline circulating cytokine levels. Those in the THC+CBD strain condition showed lower average cytokine expression levels in the blood compared to the group that used the THC strain (*F*=6.77, *p*<0.02; See Fig. 2).

## Discussion

Our data provide preliminary validation of a novel method assessing the effects of legal market cannabis strains as they are used in the real world. Our design, using detailed pharmacological assessment of cannabinoid levels in blood, demonstrated participants' adherence to their assigned strain and lends evidence to the feasibility and validity of this observational approach to studying the effects of legal market cannabis. Although this pilot study is not adequately powered to provide a comprehensive test of the effect of different strains of cannabis on measures of subjective drug desire, intoxication, and verbal recall memory function, we provide preliminary data assessing the acute effects of commercially available cannabis strains (THC and THC+CBD). With regard to these preliminary analyses, findings suggested that although the individuals using the THC+CBD strain used more of their cannabis before the experimental assessment, effect sizes indicate that use of the THC+CBD strain may be associated with lower THC blood levels. Thus, although individuals titrated up their use, potentially to compensate for lower THC strength in the strain, they did not have THC exposure levels that were higher than the THC-only group.

These preliminary data also suggest that acute use of a THC+CBD strain was associated with a lower desire to use cannabis and with feeling less mentally and physically stoned than use of the THC strain, which is consistent with several laboratory and observational studies,^14,17,35^ but inconsistent with studies that have suggested that CBD does not alter THC's positive subjective effects.^19,36,37^ The reason for conflicting findings is unknown; however, we note that the majority of studies inconsistent with our findings have either tested orally administered, often purified or synthetic, cannabinoids, or used different CBD and THC doses than are typically available and used by consumers in legal markets. The specific form and dose of CBD are important as CBD has a very complex dose–response curve,^20,38^ which may differ when CBD is administered in isolation versus by an extract that is primarily CBD, but also contains low levels of other phytocannabinoids.^20^

Thus, methodological differences across this very small literature of studies, including form, dose, and timing of administration, as well as administration of isolated versus more naturalistic sources of CBD, pose a challenge in directly comparing associations among cannabinoid exposure and acute effects found in our study to prior work. Those limitations notwithstanding, our study is the first to examine the immediate effects of CBD and THC in the context of self-administration of randomly assigned smoked flower strains consistent with legal market THC and CBD potencies and ratios. Findings extend those from a prior naturalistic study that suggested that use of strains with higher CBD content attenuated the drug reward properties of cannabis.^14^

Importantly, our findings suggesting the impact of the CBD-containing strain (THC+CBD) on lower subjective drug effects could be explained *either* by differences in THC concentrations or by differences in the presence of CBD across the two strains. However, our models testing these questions relied on quantitation of blood levels of THC and its metabolites, thus accounting for any differences in THC exposure across participants and suggesting that differences in THC strengths across the two strains cannot fully explain reported differences in subjective effects. However, there are complex interactive effects with cannabinoid strength and ratio, the individual users, and the natural environment that are unmeasured in this study. Despite these limitations of our observational design assessing the effects of legal market cannabis, these preliminary findings lend justification for further research on how strains with varied concentrations of THC and CBD cannabinoids impact the pharmacological and behavioral effects of cannabis use.

Although those assigned to the CBD-containing strain did not differ significantly from those assigned to the THC-only strain on verbal recall memory function under the influence of cannabis, this research question warrants further investigation. It is not clear whether a small, but significant, effect of CBD attenuating memory impairment would emerge in a larger sample or whether, as has been found in some previous work, use of THC, regardless of the presence of CBD, does not worsen cognitive performance in regular users due to tolerance.^19^ Pre-clinical animal^39^ and some controlled clinical studies have suggested that CBD reduces THC-related memory impairment.^35,40^ Naturalistic studies, comparing cognitive performance across individuals who use different types of cannabis, have also suggested that CBD protects against the negative cognitive effects of THC.^41^ However, prior naturalistic studies assessed users already preferring CBD-rich strains^14,42^ and did not randomly assign users to a period of use of a high CBD-based strain as done in this study.

We show also preliminary results from our exploratory aims suggesting that peripheral inflammatory markers, which may underlie physiological and medical effects of cannabis, vary based on the two strains (Fig. 2). The CBD-containing strain was associated with decreased cytokine levels, which could be explained by either the presence of CBD or differences in THC across the strains. Although preliminary, this is the first evidence that a CBD-containing strain is associated with lower circulating cytokines from a human observational trial that allows users to self-administer legal market cannabis strains. This finding is potentially relevant across several fields in that the anti-inflammatory properties of CBD are thought to underlie the potentially beneficial effects of cannabis^13–16,43^ across a variety of disease states, including potential therapeutic uses for cancer^44^ and epilepsy.^45^

In addition to sample size, a major limitation of this study is the fact that participants had to use cannabis at home and then be transported to the laboratory, due to our inability to study commercially available strains in the laboratory. We were unable to assess participants before use on the experimental day. Furthermore, because blood levels start to drop rapidly after use, variability in the time between using and the blood draw and assessments is likely to add considerable noise to the data. Future studies could utilize a mobile laboratory taken to the participants' location to decrease the amount of time between use and assessments, and still rely on legal market products. The mobile laboratory would also allow assessing participants both before and after they use cannabis, increasing experimental control. Another limitation of our design is that we were not able to observe either which strain a participant used or how much they used. Although assessing cannabinoid levels in blood attenuates these concerns to some degree, it is nevertheless possible that the participants used some other product or combination of products other than what they were assigned. Until such time as investigators can legally directly observe participant use of legal market cannabis products, this is a limitation that will be shared with all studies in this area.

## Conclusions

The findings of this study suggest that our novel methodological approach using legal market strains in ecologically valid environments (i.e., participants' homes) was valid and feasible. Analysis of cannabinoid blood levels was a key piece of our design that enhanced the feasibility and validity of our approach. Observational research using commercially available strains may provide new insights, including data on the impact of high THC strength and CBD-enriched legal strains in users, and complement more controlled laboratory studies.

## Abbreviations Used

THC: tetrahydrocannabinol
CBD: cannabidiol
DC: District of Columbia
ISLT: International Shopping List Task
TLFB: Timeline Follow-Back
POMS: Profile of Mood States
